# Lamps

**Published:** 1876-06

**Authors:** G. A. Robinson

**Affiliations:** Jackson, Mich.


### DENTAL REGISTER.

VOL. XXX.] JUNE, 1876. [No. 6.

### LAMPS.

BY G. A. ROBINSON, JACKSON, MICH.

*Delivered before the Class in Michigan Dental College.*

Lamps, commonly defined, are vessels containing combustible fluids for artificial light. Figuratively speaking, they are any kind of light in morals, science or literature. We shall use them at this time as lighted vehicles of thoughts that burn and blaze, and thus become the guide of the human heart, the human will and human action in the struggling journey of life. To say something of these lamps that are to light us through the dark hours of a professional life will be comparatively easy; but to say something new and something that has not been said before is no small task. The first and most essential for all learners is the lamp of obedience.. Obedience to the methods of study. The way and manner in which we must learn in order to a thorough understanding of the subject and this lamp must be trimmed and burning. All the great men of science have been willing to follow their favorite pursuit to its source, no matter how many crags and rocks they clambered over or how small and obscure the stream became or the distance traveled or the time consumed in reaching the desired object. Man himself is a mass of incongruities; he reflects and reasons and then follows his desires or passions. He is free in chains that bind him faster than steel. From Copernicus to Agassiz; from Luther to Channing; from Washington to Lincoln, the truly great have always been obedient.

In the far off story of the Bible we read of the disobedience of man; later, we read that the command to go through the Red Sea was obeyed by the Children of Israel before they could reach the promised land; farther along and the prophet was taken to a high mountain to curse the chosen people of the Lord. As he beheld the tents of the obedient children whiten the whole plains below him he exclaimed: "How can I curse whom the Lord has not cursed; and how shall I defy whom the Lord has not defied;" and foretold all the splendid fortunes of Israel. Paul, when before Agrippa reciting his conversion on his way to Damascus, said: "Whereupon, O King Agrippa, not disobedient to the heavenly vision, I go forward to do the will of my master." And Jesus himself said: "Father, if it be possible let this cup pass from me; nevertheless not as I will, but as thou wilt." And so we learn from the ages that are gone before us that the truly great have learned to follow this lamp of obedience that has been placed in every human heart.

After we have become obedient to learn we must next enquire *what* we are to learn, and next *when* we are to learn. We must first of all learn to subdue the body to the mind; to repair the irregularities caused by many generations of disobedience. The iniquities of our fathers have been visited upon us, and you, my young friends, must help to repair this transgression, and in your turn become teachers, and try and teach those whom you have under your charge to become obedient, even as you now try and become obedient yourselves. And you must begin now; to-morrow will be too late; to-morrow has something else for us to do. It is so in every care and duty in life; and it is so in every profession. The person who puts off a lesson or a privilege has lost it forever. The breath we drew yesterday is good for nothing for us to-day. The world is a forever pass on, pass on, this side of the grave, and a never return in Eternity.

It is useless to look after yesterday, that day has gone and passed into the eternal world. This eternal law of demand and supply of the hours and the days and the years, is to teach us vigilance and obedience to the law of labor in the demands made upon us for the good of the world, from the smallest gnat that flies, to the great beyond that is before us, it is the self same thing.

We grow in goodness and excellence from the excellence and goodness of yesterday. All the good we performed yesterday is our capital stock and trade for our labor to-day. Every tooth well filled helps fill a tooth better on the morrow. Every set of teeth well adjusted and beautifully finished helps to make better the set that is to come after it. There may be excesses of good things. Excesses in the *uses* but not in the *good* itself, as the excess of fire is a conflagration. Excess of the soft south wind is a tornado. Excess of blood in any portion of the human body is congestion; and congestion often causes death. Excess of oxygen in the air will destroy life. Excess of arsenic in destroying nerves of teeth will destroy the whole structure. Excessive finish of a filling will sometimes destroy its usefulness. I have in my mind an operator, who so excessively files and polishes the edges of his fillings that he oftentimes weakens the enamel and injures the plug that is otherwise very beautifully and skillfully made and adjusted. Hydrochloric, acetic and nitric acids may be good when used in syphilitic sore throat, but they are the conflagration to the teeth, unless administered with the greatest caution.

I shall not go into the remote cause of all our troubles, perhaps I could not tell you if I should try. We are only to take the living facts that are before us, and try and remedy the effects of some latent or unknown cause, and thus subdue these present evils by the power of good.

We must not expect to cure we can only remove the obstructions. We can not cure any more than we can make the blade of grass or of corn. We remove the stones and the weeds, and the sun light and the rain and the invisibles in the atmosphere grow the grass and the grain. This leads me to the subject of extracting the teeth. When we have extracted a tooth it is gone. It is like the dead leaf of the tree that has fallen to the ground. In the life of the tree it is always putting forth new leaves; but the withered or injured end of a single leaf is never repaired. The leaf that is dead may go to nourish the trunk, but it can never be a leaf with life again. It is not precisely so in the life and death of a tooth in any perceptible manner, although nature is always helping to restore to its natural functions whatever becomes impaired by accident or decay. One of the great duties of the dentist is to teach people to love their teeth; love them for their beauty and their utility, because people are always careful of anything they sincerely love. People who have this love of themselves will always be obedient to the demands of their natures; they will not love their mouths and teeth because they have cleaned them, but they will clean them and take care of them because they love them.

The subject of diseases of the teeth and gums deserves more than a passing notice. It has been too long neglected, and is one that will grow upon every one who has a true love for the profession. Cleanliness first, and depletion of the gums second are the safest and simplest for young practitioners. The same principle of cure applies to the filling of teeth; we only remove the obstructions and hermetically seal the orifice or opening and leave the balance to nature and the tender care of those keepers (not the owners) of these most delicate and beautiful organs. When we are asked to warrant fillings, we should ask the patient if they will agree to warrant the mouth to be clean, for on this requisition depends much of our success. All love and hence all action comes from within; so all wrong ideas are generally so grounded in our hearts that it is only by strict obedience to those who have or are supposed to have a higher and more perfect light, that we can be raised to a more elevated plain of thought and action.

The next lamp is the lamp of humility, I do not mean that fawning and sycophantic kind that is so common in the market; but a humility that will stop and help along those who are working with you, who have not had as much opportunity as has fallen to your lot before you came here as learners; those who may only have a *rush light*, while you may think you are burning *pure gas*, and that too made from better coal than your neighbor. The same sort of character necessary to this, will enable you to look after your poorest patients as well as the most wealthy. You can not tell how many hearts have bled with the desire to preserve their teeth but who have not had the money to pay for it; or how many students have had the desire to be present at this class, but whose pecuniary embarrassments would not allow it.

There has never been a great man without humility. The learned who know so much, soon begin to feel how much more there is to know that they can never find. Darwin and Tyndall and Spencer quail alike before science, but like Diogines with his lamp of humility and truth and obedience hunting for an honest man, are always seeking for the hidden things in nature not yet revealed. Jealousy and self-assurance always accompany a second class intellect. Many men who can talk well, think very little, and a far greater number there are who absolutely fail to perceive the truth from lack of obedience and humility. The next lamp we have to deal with is the lamp of *truth*, absolute truth, as nearly as can fall to the lot of mortals. We must be true to ourselves, so we may be true to every one with whom we become acquainted or associated. This is the pure gold in the nugget, this is the clean face of the coin. In the hand, in the instrument, in the head, in the heart, in the life, it is the twin sister of consciousness. Its fillings never grow black and leak, its fangs are never left in the jaws, its plates are always made to fit without cavil or equivocation; sets of teeth always articulate and are adapted in the best possible manner; it never underbids a fellow dentist; it never does work without a reasonable compensation, or charges an exorbitant price, because it has the power leagued with the opportunity; it never insinuates that its neighbor is no better than he ought to be; it never takes praise where it is not merited, or stands quietly by and hears a slander against the weak or the innocent, who are absent, without a reply.

In the day or in the night,
In the dark or in the light,
In the sun or in the shade,
Its beauties never fade.
Without boldness, without fear,
It is never insincere;
In the wet or in the dry,
It looks up towards the sky;
In the rich and in the lowly,
In the sacred, in the holy,
In the peace and in the war,
It's voice is heard afar,
Saying, Truth, thou blessed son,
With thy race so well begun,
We hail thy advent from above,
Born of the highest, purest love.

The next must be the lamp of *Sincerity*. No person has ever arrived at any degree of excellence who has been insincere. The politician who follows the crowd; the soldier who fights for wages; the minister who preaches what he scarcely believes; the merchant who urges the buying of an inferior article; the doctor who calls when he knows you have no need of medicine; the lawyer who urges you to carry on a suit where he feels you are in the wrong; the dentist who performs operations contrary to his highest judgment, because there is a dollar for his pocket or to please some whimsical person who is ignorant of the laws of our highest nature. Any of these persons may have a grand dwelling or a fine office or be received into good society, but they are as far from sincerity as a man who forges a note or breaks into your house in the night time. But the most deplorable part of the insincere man is that he always feels that the world does not do him justice, that he is slighted in society and by his fellows, and that in this world fight he must contrive somehow to get square with his neighbors; he adopts as his motto the old Italian proverb, "that if you would succeed you must not be too honest and too good." This is the great rock on which he is wrecked in his life's work, in his influence in society and in the world.

Another lamp is the lamp of industry, and another is the lamp of persistency. As persistency pertains more immediately to acts, and truth to the speech; as sincerity is identified particularly with what we do, and truth to what we say, so industry belongs to the commence of life's duties and persistency to the finish. This is of the greatest importance to true manhood, and is indispensable to the good dentist. I know many industrious persons who never have any one thing completed; so I know of many dentists of the same character, a thousand things on hand and well begun, but not one that is ever ended. Men of this character never have a complete and rounded life. A dozen books begun that they know very little about, a dozen pieces of work on hand that have been called for a dozen times, a dozen patients disappointed day after day and week after week. These little unimportant and all important acts, or lack of acting, wound sincerity, murder truth, crush humility and deal a death blow at obedience. Persistency is like a tree that is always green and never loses its leaves, or a flower that is forever in blossom and never loses its fragrance. Persistency is the leading trait in the mother in training the child, and of the soldier in winning a battle. You may be slow, you may be quick, you may be patient, you may be irritable, you may be dull and stupid or you may be brilliant, you may have genius or you may have memory; but without persistency you fail, for it is the foundation stone of success, and when coupled with love is the corner stone of self-sacrifice. It is the crucible in which all the other faculties are fused, it is the sun without a cloud, it is the day without a night, it is the lamp that never grows dim, for the oil is drawn from the deepest recesses of the human soul.

The lamp of politeness should not be overlooked in the practice of our profession. Every person should know the importance of this element of character for a sure success. The work we are called upon to do is necessarily unpleasant, and while it is sad to cause pain in any manner, it is necessary to inflict it upon some unfortunate almost every day. I do not mean by politeness a sickly sentimentalism that so often passes for that, among shallow people, but a kind attention and assistance to those placed under our charge; a sympathy with their suffering that is the companion of patience; a politeness that will inspire confidence and regard for our wishes; a politeness that is as commanding and graceful as the south wind is commanding to the waving pine, or the flower that blossoms under the soft influence of the sun; a politeness that will make your patients your dearest friends; a politeness that is so simple that all people will understand that it is genuine and unaffected; a politeness that will command the respect of all true men and women; a politeness that will drive the vulgar and profane from your presence and give you a passport to true society. You may be well behaved without culture but you can not be polite. Culture is the foundation of true politeness. With manners all your own, with awkwardness of arms and of feet, but with tenderness of manners and culture, you can not be otherwise than a polite gentleman. Politeness is never too bold; it is never overbearing or assuming; it is not arrogant or puffed up; it is the counterpart of goodness and the twin sister of self-sacrifice; it apologizes for blunders and mistakes, and it is the companion of forgiveness. Politeness may often succeed where strength of character will fail, but a combination of both is a sure guarantee of success. And lastly you must have the lamp of adaptation. The dentist can never know exactly what he is to be called upon to do. As our faces all resemble each other and are still unlike, so every operation, while somewhat the same, is always different. What we should be able to do is to perceive what is necessary to perform an operation at first sight, and do what we think ought to be done as quick as thought. Adaptation gathers all the forces at command and hurls them with precision at the right point; it is the commanding general of all our faculties; it is the Napoleon of all our battles; it suggested the rubber dam to Barnum and gave the battering ram of the mallet and the polishing anvil to Atkinson; it gave the engine to Morrison and White and the ultimatum of chairs to Perkins; it gave the pure soft foil to Bull and Abbey and the adhesive qualities to Watts and his disciples; it has advanced the profession from the gold to the rubber for the million, and given us the celluloid to beat Bacon; it is the lever and the screw of all the mechanical appliances, and it is the philosophy, chemistry and therapeutics to renovate the oral cavity of the world; it adapts the instrument to the cavity and the gold to the instrument; it substitutes the plaster for the wax and gave us the impression cup for the plaster. It is almost genius but it is not genius, for it never invents; it is only suggestive and methodical; it uses every thing that genius has invented, but it has never yet taken out a patent; it is the thoughtful man without stopping to think, and it is the student without waiting to study. In short it is the American dentist, the man who has made dentistry from a small beginning to a great and learned profession; the man who has elevated an art until it has schools and colleges and become the peer of many older professions in thought and activity and in the life of its members. If we would be good dentists we must in the beginning be good men. Good operators may have sometimes been demoralized, but there has never been one thoroughly good one who has been inwardly a very bad man.

Our profession began with a few straggling itinerants, who were scorned by the medical profession and held in contempt by a large portion of the human family. But by perseverance, study and scientific research, respectability of character and culture of those who have engaged in it as a life's work and a labor of love, it has colleges and schools, and teachers now who are the peers of the other learned professions. If those of you who are here as learners would wear the laurels already won, and to be handed to you by those who are assisting you to become strong you must accept it as a life work and a labor of love.

If we would be really great it is necessary that all the lamps that I have enumerated should be lighted in our hearts, with many others not mentioned. That will be necessary to give you a rounded life that will be one great blaze of light to illuminate the world. All our life that has been lighted by these lamps of love must be given to every operation, for our love is life and our life is only love. Every act for the good of our fellows is begun and finished in love, and this is the love and life that is given to us from God. In the silent hours of the night-time his great hand is the shuttle that weaves the life into our bodies with every breath we draw, as the lightning is woven in the clouds before the rain. In this great ocean of humanity the good acts we perform are the flakes of snow, or the finest mist, or the infinitesimal dew that waters and fertilizes the world. The dentist must be a man of large capacity and large charities; the world makes certain levies upon him for the privilege of living in it. He is expected to do fully his part for the advancement of civilization, the amelioration of society and the building of towns and cities. He must work outside of himself, or the pale of selfishness, and for the love of doing good. He must be actively good, the passively good man is a selfish man. His arguments are always why he should not do, instead of why he should do. He loves his family because it is part of himself; he goes to church because it is a good insurance investment. If the moral law is written in his heart the weeds of selfishness choke it so that it never can act; whenever he is solicited to give, or to do any work of charity, he has always given so much, or is too busy, and contents himself with the motto that "charity begins at home." Not so with the great man for he is always the good man, he reasons from another standpoint. He always argues why he should give and do, instead of why he should not do. His walk among his fellows is so quiet that you will not notice him until you look for him to act. He is too busy to ride so he always goes on foot; he performs kind acts as he breaths, without anybody knowing it but himself. He believes with all his heart and has the ability to make others believe without reflection what he believes, because of great reflection.

This world is a step ladder and when we take the first step up, there is an abyss below, but there is a height above to which the good always aspire. The great men are those who can carry the most dead weight of their fellows to the top without stumbling. He is a religious man though perhaps he is never seen inside of a church; he is a poet, though perhaps he has never written a line; his intellect and moral sentiments exactly balance; he turns all thoughts into things for the good of his fellows, while the selfish man turns all things into thoughts for his own agrandizement. Let us all try and be good men.

Every achievement is accomplished by the light of the lamps of faith and love; every thought is a manifestation of nature through man. The protoplasm is in the infinite, so every experience is contact with some form of nature in daily life. It is only those who are able to divide and subdivide that succeed, we can not master the united whole.

Napoleon gained his battles by division; he cut his enemies in twain and subdued them one half at a time, as the beam of a steam ship falls down and divides the waters that we may cross the sea, or the plow divides the land that we may grow the grain. Man who knows the most is always making blunders; nature is always true and upright; man is constantly falling, nature gives a blow for every disobedience, and often leaves him to guess the cause; hence comes the necessity of the professions. Ours among the most useful and ornamental came almost last; faith in the ability to correct the mistakes, and love of harmony instead of disease and deform has created the dentist. Whenever we perform acts of duty it is always the love of duty and faith in the right that prompts us to act, and these qualities must first of all be within ourselves, as much as the love of the rainbow or the sunset; almost every person possesses these loves as much as the love of food and home. Love of duty is rarely enthusiastic, it grows out of a passive state of rectitude; it is the lowest form of faith and love. Every known form of instrument was enthusiastically born in "Corydon Palmer;" he saw the curves and the angles in the oaks and the willows; he saw the most beautiful and polished fillings in the flower garden before the door and these he transferred to the mouths of his patients to be the examples to the world.

Passive duty always has a dull instrument, oftentimes a dirty and sluttish office; has an uncombed head and dirty hands. It is water in the pool, but it is the abode of frogs and lizards and unfit for use until it has been cleansed. This indifference has no reward; it is like the potato growing in the cellar, or the flower that is blossoming under the crag. There can be no excess of love or faith, in proportion as they are large and grand they have compensation; as the man who studies botany or astronomy finds a new world and a new firmament, so the man who loves his profession finds in every new achievement a new satisfaction and delight. Every person should be more of a man for being a dentist, he is constantly exercised with the love of the beautiful, the beautifying process is always before him. He is associated with the better part of humanity in his daily life. More than any other profession he can say confidently to the ills of those with whom he has intercourse, "Get thee behind me." But with all the advancement we have made we must suppose that dentistry is in its virgin life; the organs of mastication must be brought back to their primeval beauty and usefulness and this is being gained day by day. The failures of the past fifty years are being swept away as by a flood; the ethics of the profession are just being understood. Often out of our mistakes has blossomed a flower that will never die. Dentistry was once considered a pretension, now it is admitted an operation for the benefit of the world; and this has grown out of the lighted lamps of faith and love in the hearts of the members. Faith and love are the unconscious action of the soul; they grow with our knowledge and experience with things, but they are founded in the desire to reform, utilize and beautify those freaks in nature that have been marred by accident or decay. We can not tell what aids science may bring along to prevent, but the chief business of the practitioner of to-day is mainly to repair and regulate; to take the highest type and try and restore the unfortunate to their normal condition. To do this is the culmination of good for the present.

The ultimate is what we may be and become in the future; that is, to prevent the accident or the disease. As the bended tree when it is riven by the wind, is helped by the forces in nature to become erect and upright, so it should be the business of the dentist of the future to restore to the mouth its original beauty and usefulness and thereby prevent the calamity that is now abroad in humanity and the world.

